# Responsiveness of the short-form health survey and the Parkinson’s disease questionnaire in patients with Parkinson’s disease

**DOI:** 10.1186/s12955-017-0642-8

**Published:** 2017-04-18

**Authors:** Xiao-Jing Tu, Wen-Juh Hwang, Shih-Pin Hsu, Hui-Ing Ma

**Affiliations:** 1 Institute of Allied Health Sciences, College of Medicine, National Cheng Kung University, 1 University Road, Tainan, 70101 Taiwan; 2 Department of Neurology, College of Medicine, National Cheng Kung University, 1 University Road, Tainan, 70101 Taiwan; 3 Department of Neurology, E-Da Hospital, I-Shou University, 1 Yida Road, Yanchao District, Kaohsiung, 82445 Taiwan; 4 Department of Occupational Therapy, College of Medicine, National Cheng Kung University, 1 University Road, Tainan, 70101 Taiwan

**Keywords:** Health-related quality of life, Parkinson’s disease, PDQ-39, SF-36, Responsiveness

## Abstract

**Background:**

The responsiveness of a measurement instrument is important for understanding its ability to detect changes in the progression of a disease. We examined and compared the internal and external responsiveness of the 36-item Short-Form Health Survey (SF-36) and the 39-item Parkinson’s Disease Questionnaire (PDQ-39) in patients with Parkinson’s Disease (PD).

**Methods:**

Seventy-four patients with PD were evaluated using the SF-36 and PDQ-39 at baseline and again after one year. In addition, their motor signs, motor difficulties of daily living, and depressive symptoms were assessed as external criteria. The internal responsiveness was examined using effect size, standardized response mean, and the Wilcoxon signed rank test. The external responsiveness was examined using receiver operating characteristic curves, correlation analyses, and regression models.

**Results:**

Both instruments were partially sensitive to changes during the 1-year follow-up and able to discriminate between patients with improved versus deteriorated motor signs. In addition, both were similarly responsive to changes in the motor difficulties of daily living; the SF-36 appeared to be more sensitive than the PDQ-39 to changes in depressive symptoms.

**Conclusions:**

The SF-36 and the PDQ-39 were acceptably internally and externally responsive during the 1-year follow-up.

**Electronic supplementary material:**

The online version of this article (doi:10.1186/s12955-017-0642-8) contains supplementary material, which is available to authorized users.

## Background

Parkinson’s disease (PD) is a progressive neurodegenerative disorder; therefore, measuring the patient’s health-related quality of life (HRQoL) to reflect the progression of PD is important. HRQoL refers to the status of one’s health in the physical, emotional, and social well-being [1]. Soh et al. [2] reported that the 36-item Short-Form Health Survey (SF-36) is the most frequently used generic HRQoL instrument, and that the 39-item Parkinson’s Disease Questionnaire (PDQ-39) is the most frequently used disease-specific HRQoL instrument. Both measures have good internal consistency, stability, and discriminant validity [3, 4], and both are recommended by the Movement Disorder Society [4, 5].

The responsiveness of a measurement instrument is important because it reflects the extent to which the instrument can detect changes in the progression of a disease and whether it can show longitudinal validity [6, 7]. Two major types of responsiveness have been recommended: internal and external [8]. Internal responsiveness refers to the ability of an instrument to detect change at two different time points. External responsiveness refers to the ability of an instrument to change relative to the change of a reference measure. These two types of responsiveness provide different but complementary information: internal responsiveness reflects patient-level changes, and external responsiveness reflects a different view of patient status using another available measure. Although the responsiveness of HRQoL instruments has been examined in several studies [9, 10], few have examined the comparative responsiveness of the SF-36 and the PDQ-39 in patients with PD. Schrag et al. [11] reported that the PDQ-39 was acceptably responsive to small changes in PD progression during a 1-year follow-up, independent of any intervention or external measure. Using self-reported changes in health status as external criteria, Brown et al. [12] reported that the SF-36 was more responsive than was the PDQ-39 in an 18-month follow-up. However, neither study [11, 12] included clinician-judged motor evaluation or psychological measures as external criteria to evaluate the responsiveness of the HRQoL instrument.

We assessed and compared the internal and external responsiveness of the SF‐36 and PDQ-39 in patients with PD. Evaluations of motor signs and motor difficulties of daily living and a measure of depressive symptoms were used as external criteria. Many interventions for patients with PD have targeted motor problems and depression [13, 14], but few have examined the comparative responsiveness of the SF-36 and the PDQ-39 against these external criteria to determine which one to use in longitudinal outcome research in patients with PD.

## Methods

### Study design and participants

Between September 2014 and August 2015, participants were consecutively recruited from the neurology departments of two medical centers in southern Taiwan. The inclusion criterion was a diagnosis of PD by a neurologist. The exclusion criterion was a Saint Louis University Mental Status Examination (SLUMS) [15] score that indicated severe cognitive impairment (a score < 19 for people with less than a high school education and < 20 for those with a high school education or above). The SLUMS is a 30-point questionnaire used to screen for cognitive impairment; it includes items for orientation, memory, attention, and executive function. Participants were concurrently interviewed and evaluated face-to-face at baseline and after 12 months of follow-up. The notion that a 1-year follow-up was sufficient was based on a prior study [16]. Most (80.4%) evaluations were carried out when the participants were in the “on” phase of the medication cycle.

This study followed the principles of the Declaration of Helsinki. The study protocol was approved by the Institutional Review Board of National Cheng Kung University Hospital (B-ER-101-171) and the Institutional Review Board of E-Da Hospital (EMRP-102-024). Written informed consent was obtained from each participant.

### Measures

HRQoL was examined using the SF-36 and PDQ-39. The SF-36 [17] includes eight domains: physical functioning, role limitations caused by physical problems, bodily pain, general health perceptions, mental health, role limitations caused by emotional problems, social functioning, and vitality. Each domain score and the summary score range from 0–100, with higher scores indicating a better HRQoL. The Taiwanese version of the SF-36 has been tested in middle-aged women [18] and stroke patients [19] and has been reported to be reliable and valid.

The PDQ-39 [20] has eight domains: mobility, activities of daily living, emotional well-being, stigma, social support, cognition, communication, and bodily discomfort. It is composed of a summary index and eight domain scores, each of which ranges from 0 to 100. A higher score indicates a more frequent self-perceived difficulty in HRQoL. The Taiwanese version of the PDQ-39 has been reported to be reliable and valid in patients with PD [21].

Parts II and III of the Movement Disorder Society Revision of the Unified Parkinson’s Disease Rating Scale (MDS-UPDRS) [22] and the Geriatric Depression Scale (GDS) [23] were used as external criteria. The MDS-UPDRS Part II includes 13 items that assess motor difficulties of daily living, and Part III contains 18 items that examine motor signs. Each item uses a 5-point Likert scale (0 = “no problems identified” and 4 = “severe problems identified”), and item scores are summed for the total score of each part [22]. The 30-item GDS is a yes/no screening scale for depressive symptoms. The GDS has good psychometric properties and has been reported as efficient in patients with PD [24, 25].

According to Revicki et al. [26], the external criteria for assessing the responsiveness might be patient-rated global improvement, clinical measures with established responsiveness, or some combination of clinical and patient-based outcomes. Because it is important to select external criteria that are relevant for the disease indication and to use multiple independent external criteria, we chose the MDS-UPDRS and GDS, which are both recommended by the Movement Disorder Society [22, 24] and frequently used for evaluating motor function and depression symptoms in patients with PD. In addition, the minimal clinically important difference (MCID) has been established for the MDS-UPDRS Part III, which served as a reference for evaluating responsiveness of HRQoL measures in the present study.

### Statistical analysis

We used the MCID of the MDS-UPDRS Part III [27] as a criterion for classifying patients with PD into Improving, Stable, and Worsening groups during the 1-year follow-up. Patients with MDS-UPDRS Part III change scores < −3.25 were considered improving, and those with change scores > 4.63 were considered worsening. The internal responsiveness of the SF-36 and PDQ-39 was then calculated using effect size (ES), standardized response mean (SRM), and the Wilcoxon signed rank test [8]. The latter was used because the change scores of the SF-36 and PDQ-39 did not follow the normal distribution. Significance was set at *p* < 0.05.

ES is a ratio of mean change scores between the baseline and follow-up measure divided by the standard deviation (SD) in the baseline measure. The SRM is the mean of the differences between the baseline and follow-up, divided by the SD of the changed scores. Negative values in the ES and in the SRM represent a worse HRQoL and positive values represent a better HRQoL. In the ES and SRM, a value of 0.2 represents a small sensitivity to change, 0.5 a moderate sensitivity, and 0.8 a large sensitivity [8].

External responsiveness was evaluated using receiver operating characteristic curves (ROCs), correlation analyses, and regression models. The ROCs were used to assess the ability of a measure to reflect a change or lack of change in the external criteria [8]. The area under the ROC is calculated for the range from 0.5 (not accurate for distinguishing improvers from non-improvers) to 1.0 (perfectly accurate) [28] based on the external criteria. When calculating the ROC, the MCID of the MDS-UPDRS Part III was used to judge whether a patient improved, remained stable, or worsened. Correlation analyses of the change scores between the HRQoL measures and the GDS and MDS-UPDRS Part II were done using Spearman’s rank-correlation. Correlation coefficients of 0.25-0.49, 0.5-0.74, and ≥ 0.75 show small, moderate, and strong associations, respectively [29]. Simple linear regressions were used for a relative estimate of the degree of variance in the change of the external criterion that could be explained by the change score of the HRQoL instrument. SPSS 17.0 was used for statistical analyses (IBM, Chicago, IL, USA).

## Results

At the 1-year follow-up, we had completed assessing 74 of the 95 enrolled patients with PD. Twenty-one (22.1%) patients were lost to follow-up: 2 had died, 7 could not be contacted, and 12 had dropped out. The baseline demographics and evaluation scores were not significantly different between the 74 patients who completed the study and the 21 who did not. Most of the patients were male (67.6%) and at Hoehn and Yahr stages I and II (75.6%) at baseline (Table 1). One year of follow-up showed that the severity of the PD and motor signs of the 74 patients had significantly increased.

**Table 1:** Demographic characteristics in PD patients (*N* = 74)

Variables	Baseline Mean ± SD (n [%])	Follow-up Mean ± SD (n [%])
Gender		
Male	50 (67.6%)	
Female	24 (32.4%)	
Age (years)	64.82 ± 9.81	
Age at Onset	58.97 ± 10.25	
Disease Duration (years)	5.85 ± 4.33	
Hoehn & Yahr Stage^a^		
I	37 (50.0%)	27 (36.5%)
II	24 (32.4%)	29 (39.1%)
III	9 (12.2%)	14 (19.0%)
IV	4 (5.4%)	4 (5.4%)
SLUMS	24.62 ± 3.43	24.16 ± 3.27
GDS	7.23 ± 6.70	8.23 ± 6.39
MDS-UPDRS Part II	8.23 ± 5.14	9.31 ± 6.40
MDS-UPDRS Part III^a^	22.22 ± 12.70	27.15 ± 13.09
SF-36 total scores	64.22 ± 20.63	61.15 ± 18.79
PDQ-39 SI	15.60 ± 11.72	18.07 ± 13.09

*SLUMS* Saint Louis University Mental Status Examination, *GDS* Geriatric Depression Scale, *MDS-UPDRS* Movement Disorder Society Revision of the Unified Parkinson’s Disease Rating Scale, *SF-36* 36-item Short Form Health Survey, *PDQ-39* 39-item Parkinson’s Disease Questionnaire Single Index, *SI* summary index, *SD* standard deviation

^a^Significant differences between baseline and follow-up using Wilcoxon test

The MCID of the MDS-UPDRS Part III showed that 16 of the 74 patients had improved and that 34 were worsening (Table 2). The improved group had significant differences between baseline and follow-up scores in the SF-36 general health and PDQ-39 mobility domains. The worsening group had significant differences between baseline and follow-up scores in the SF-36 social functioning, vitality, and total scores, and in the PDQ-39 emotional well-being, social support, communication, bodily discomfort, and summary index scores. Moderate to large responsiveness (ES or SRM > 0.5) was found for the SF-36 general health (ES = 0.88, SRM = 0.65) and PDQ-39 mobility (SRM = 0.72) domains in the improved group, and for the PDQ-39 social support (ES = −1.20, SRM = −0.51) and communication (SRM = −0.55) domains, and for the summary index (SRM = −0.55) in the worsening group.

**Table 2:** Internal responsiveness of HRQoL in improved and worsening patient groups

HRQoL domains	Improved^a^ (*n* = 16)					Worsening^a^ (*n* = 34)				
	Baseline mean (SD)	Follow-up mean (SD)	ES	SRM	*p*	Baseline mean (SD)	Follow-up mean (SD)	ES	SRM	*p*
SF-36										
Physical functioning	70.00 (25.36)	78.13 (23.09)	0.32	0.44	0.129	70.29 (23.39)	63.97 (20.55)	−0.27	−0.31	0.094
Role-physical	53.13 (46.44)	56.25 (41.33)	0.07	0.07	0.832	44.85 (40.26)	39.71 (37.50)	−0.13	−0.16	0.379
Bodily pain	74.25 (27.23)	72.19 (24.85)	−0.08	−0.15	0.611	73.44 (25.17)	67.91 (26.22)	−0.22	−0.26	0.061
General health*	51.75 (15.64)	65.56 (20.03)	0.88	0.65	0.017	52.41 (20.42)	48.88 (21.14)	−0.17	−0.19	0.271
Mental health	70.50 (19.09)	73.00 (17.44)	0.13	0.12	0.505	71.76 (23.88)	68.12 (20.62)	−0.15	−0.17	0.155
Role-emotional	60.42 (40.77)	62.50 (29.50)	0.05	0.05	0.888	58.82 (42.69)	50.98 (41.22)	−0.18	−0.19	0.388
Social functioning^†^	71.09 (29.13)	80.47 (21.39)	0.32	0.34	0.190	71.32 (24.72)	61.76 (31.07)	−0.39	−0.39	0.048
Vitality^†^	61.88 (20.89)	61.56 (20.06)	−0.01	−0.02	0.813	60.15 (23.73)	52.35 (20.61)	−0.33	−0.40	0.035
Total scores^†^	64.13 (22.65)	68.71 (18.71)	0.20	0.30	0.191	62.88 (21.54)	56.71 (19.81)	−0.29	−0.43	0.017
PDQ-39										
Mobility**	22.66 (24.60)	14.69 (18.97)	0.32	0.72	0.010	22.57 (23.82)	29.2 (24.52)	−0.28	−0.27	0.098
ADL	17.45 (18.77)	12.76 (16.42)	0.25	0.43	0.113	15.32 (16.69)	18.26 (18.46)	−0.18	−0.16	0.211
Emotional well-being^†^	10.68 (12.73)	9.64 (16.30)	0.08	0.12	0.159	18.14 (19.65)	22.67 (18.55)	−0.23	−0.26	0.035
Stigma	13.28 (18.38)	7.81 (12.18)	0.30	0.27	0.236	20.59 (22.64)	23.53 (22.62)	−0.13	−0.17	0.154
Social support^††^	8.59 (16.28)	7.03 (16.51)	0.10	0.09	0.932	4.78 (8.59)	15.07 (21.05)	−1.20	−0.51	0.001
Cognitions	12.89 (14.34)	16.02 (16.61)	−0.22	−0.19	0.429	24.26 (20.46)	29.41 (15.58)	−0.25	−0.27	0.071
Communication	6.25 (9.86)	10.94 (13.85)	−0.48	−0.34	0.172	11.03 (19.43)	20.59 (21.73)	−0.49	−0.55	0.003
Bodily discomfort^†^	21.88 (20.16)	14.06 (14.82)	0.39	0.47	0.078	21.57 (20.73)	27.45 (24.41)	−0.28	−0.32	0.049
SI^††^	14.21 (9.52)	11.62 (11.72)	0.27	0.37	0.074	17.28 (14.22)	23.28 (14.58)	−0.42	−0.55	0.004

*SF-36* 36-item Short Form Health Survey, *PDQ-39* 39-item Parkinson’s Disease Questionnaire Single Index, *ADL* activities of daily living, *SI* summary index, *SD* standard deviation, *ES* effect size, *SRM* standardized response mean

^a^The minimal clinically important difference (MCID) of the Movement Disorder Society Revision of the Unified Parkinson’s Disease Rating Scale Part III was used to classify PD patients into improved and worsening groups

*Significant differences between baseline and follow-up in the improved group by using Wilcoxon test, *p < 0.05, **p < 0.01

^†^Significant differences between baseline and follow-up in the worsening group by using Wilcoxon test, ^†^ *p* < 0.05, ^††^ *p* < 0.01

The values of the area under the ROCs indicated that both the SF-36 and the PDQ-39 discriminated between patients with improved and with worsening motor signs (Table 3). In addition, the change in the MDS-UPDRS Part II scores between baseline and follow-up had similar degrees of association with the changes in the SF-36 (*r* = −0.40, *p* < 0.01) and PDQ-39 (*r* = 0.45, *p* < 0.01) scores. The changes in the GDS scores were moderately associated with the changes in the SF-36 (*r* = −0.53, *p* < 0.01) scores and only slightly associated with the changes in the PDQ-39 (*r* = 0.29, *p* < 0.05) scores.

**Table 3:** External responsiveness of HRQoL in PD patients (*N* = 74)

Changed HRQoL scores	AUC^a^ (95% CI)			Correlation Coefficient^b^		Regression Model			
	Improved vs. stable	Worsening vs. stable	Improved vs. worsening	MDS-UPDRS Part II	GDS	MDS-UPDRS Part II		GDS	
						β	R^2^	β	R^2^
SF-36	0.656 (0.48 − 0.83)	0.543 (0.39 − 0.70)	0.695 (0.54 − 0.85)	−0.40**	−0.53**	−0.15	0.21***	−0.18	0.23***
PDQ-39	0.680 (0.51 − 0.85)	0.625 (0.48 − 0.77)	0.754 (0.61 − 0.90)	0.45**	0.29*	0.25	0.26***	0.17	0.09*

*SF-36* 36-item Short Form Health Survey, *PDQ-39* 39-item Parkinson’s Disease Questionnaire, *MDS-UPDRS* Movement Disorder Society Revision of the Unified Parkinson’s Disease Rating Scale, *GDS* Geriatric Depression Scale, *AUC* area under the curve, *CI* confidence interval

^a^External criterion is the MDS-UPDRS Part III; ^b^Used Spearman’s rank-correlation coefficients

*p < 0.05, **p < 0.01, ***p < 0.001

Regression analyses showed that the changes in the SF-36 and PDQ-39 scores explained more than 20% of the variance in the change scores of the MDS-UPDRS Part II (Table 3). A one-unit change in the SF-36 and in the PDQ-39 scores, on average, corresponded with approximately −0.15 and 0.25 points, respectively, in the changes in the MDS-UPDRS Part II scores. In addition, the change in the SF-36 scores explained 23% of the variance in the change scores of the GDS. A one-unit change in the SF-36 score corresponded with a change of approximately 0.18 points in the GDS score.

## Discussion

We examined the internal and external responsiveness of the SF-36 and PDQ-39 in a 1-year follow-up of patients with PD. We found that both HRQoL instruments partially detected changes in patients with improved and worsening motor signs, and were able to discriminate between them. In addition, both instruments were similarly responsive to changes in the motor difficulties of daily living, and the SF-36 was more sensitive to change in depressive symptoms.

Our findings support the longitudinal validity of the SF-36 and PDQ-39 for measuring the long-term health outcomes of patients with PD. However, our finding that the PDQ-39 was as responsive as the SF-36 for detecting 1-year change is inconsistent with Brown et al. [12]. The inconsistency might be attributable to the different criteria used: they used a subjective rating of overall HRQoL, but the present study used a clinician-judged evaluation of motor signs. The internal responsiveness of the HRQoL measures also seemed to depend upon the criteria used.

We examined the internal responsiveness of the domain scores to better understand the characteristics of these two HRQoL instruments. The significant and moderate changes in the social support and communication domains of the PDQ-39 in the worsening group signal the continuous decline in social interaction associated with worsening motor signs in patients with PD. The motor signs evaluated in the MDS-UPDRS Part III included speech, facial masking, rigidity, bradykinesia, gait, posture, and tremor. A quantitative study [30] reported motor signs as a significant predictor of communication in the PDQ-39. In addition, much qualitative research [31–33] has vividly described how patients with PD perceived their movement and communication difficulties to be distressing and socially embarrassing. Such stigmatized feelings might subsequently lead to decreased social interaction. Additional research is needed to quantitatively examine the relationships between motor signs, experienced stigma, and social interaction in patients with PD.

The results of the internal responsiveness of the two HRQoL scales might be related to the type of anchor used, the characteristics of the HRQoL scales, and the changes that occurred in our patients. We used the MICD of the MDS-UPDRS Part III as a criterion for classifying patients into improving, stable, and worsening groups. The *post hoc* calculation of the change scores of the other measures for these three groups indicated that while the stable group had smaller changes in the MDS-UPDRS Part II and PDQ-39 than did the other two groups, they had substantial changes in the GDS and SF-36 that were comparable to those of the other groups (Additional file 1).

Moreover, when we calculated the changes in domain scores (Additional file 2), we found that the stable group had smaller changes than did the other two groups in the SF-36 domains of physical functioning and role-physical, and in the PDQ-39 domains of mobility and ADL. In contrast, the stable group had a notable decline in the SF-36 domains of general health and role-emotional, and in the PDQ-39 domain of emotional well-being, which might be associated with increased depression as measured by the GDS.

Overall, the heterogeneous results among the domains of the HRQoL scales reflect the complex interplay between the physical, psychological, and social factors of an individual. Using the MDS-UPDRS Part III as the anchor has the strength of distinguishing those with changed motor signs, but the results might not be generalizable if other anchors (e.g., GDS) are used.

Using the MCID of the MDS-UPDRS Part III as a gold standard for evaluating external responsiveness, we found that both the SF-36 and the PDQ-39 discriminated between patients with improved and with deteriorated motor signs. In addition, when the MDS-UPDRS Part II was used as the external criterion, the SF-36 and PDQ-39 had similar degrees of sensitivity to changes in the motor difficulties of daily living. Our *post hoc* correlation analyses of the change scores between the HRQoL domains and the MDS-UPDRS Part II showed significant correlations in the SF-36 physical functioning, bodily pain, social functioning, and vitality domains (*rs* = −0.233 ~ −0.487) and significant correlations in the PDQ-39 mobility, activities of daily living, emotional well-being, social support, and bodily discomfort domains (*rs* = 0.300 ~ 0.375). This suggests that the motor difficulties of daily living in patients with PD contribute to compromised HRQoL across physical, psychological, and social domains.

When the GDS was used as an external criterion, the SF-36 appeared to be more responsive to changes in depressive symptoms than did the PDQ-39. This might be because the SF-36 has three domains (mental health, role-emotional, and vitality) that ask questions directly related to psychological health, while the PDQ-39 has only one (emotional well-being). Our *post hoc* correlation analyses of the change scores between the HRQoL domains and the GDS also found that SF-36 had more domains significantly correlated with the GDS than did the PDQ-39. Significant correlations were found in six of eight domains in the SF-36 (physical functioning, role-physical, general health, mental health, role-emotional, and vitality; *rs* = −0.237 ~ −0.532), while in the PDQ-39, significant correlations were found in only three of eight domains (mobility, emotional well-being, and social support; *rs* = 0.285 ~ 0.316).

It is noteworthy that while the change scores of the SF-36 domains were significantly correlated with the change scores of either the MDS-UPDRS Part II or the GDS, some domains (stigma, cognition, and communication) of the PDQ-39 were correlated with neither one. Because the PDQ-39 items were generated from in-depth interviews with patients as concerns of the effect of PD, future research should include other external criteria (e.g., social interaction) to validate these unique domains.

This study has some limitations. First, we used convenience sampling and recruited our participants from two medical centers. The generalizability of our findings might thus be limited to clinic-based patients with PD. In addition, 22.1% of participants at baseline were lost to the 1-year follow-up. Although their baseline demographics and evaluation scores were not significantly different from those of the patients who completed the study, the smaller sample size might affect the validity of our results and conclusion. Moreover, all patients were tested at a single point in time without controlling for the medication cycle phase during the interview or evaluation. We allowed medication effects to vary naturally, because the purpose of this study was not to test responsiveness to any intervention. However, this design might lead to some confounding of the evaluation results. Future research probably should control for participants’ medication status to arrive at a more precise estimation of intervention effectiveness. Finally, psychometric testing of the Taiwanese version of the SF-36 has been done only in middle-aged women and stroke patients [18, 19]. Future research probably should examine the psychometric properties of the SF-36 in Taiwanese patients with PD.

## Conclusions

This is the first study that uses a clinician-judged evaluation of motor signs and a psychological measure as external criteria to evaluate the internal and external responsiveness of HRQoL measures in patients with PD. Overall, both the SF-36 and the PDQ-39 were partially sensitive to change in the 1-year follow-up and discriminated between patients with improved and with deteriorated motor signs. In addition, the SF-36 and the PDQ-39 were similarly responsive to changes in the motor difficulties of daily living, and the SF-36 was more sensitive than was the PDQ-39 to changes in depressive symptoms.

## Additional files

Additional file 1: Changed scores of measures in three groups. (DOCX 17 kb)

Additional file 2: Internal responsiveness of HRQoL in the stable patient group. (DOCX 20 kb)

## Abbreviations

GDS: Geriatric depression rating scale
HRQoL: Health-related quality of life
MCID: Minimal clinically important difference
MDS-UPDRS: Movement Disorder Society Revision of the Unified Parkinson’s Disease Rating Scale
PD: Parkinson’s disease
PDQ-39: 39-item Parkinson’s disease questionnaire
ROC: Receiver operating characteristic curves
SF-36: 36-item short form health survey
SRM: Standardized response mean
