# Supplementary material for: Responsiveness of the short-form health survey and the Parkinson’s disease questionnaire in patients with Parkinson’s disease
Source: Health Qual Life Outcomes. 2017 Apr 18;15:75. doi: 10.1186/s12955-017-0642-8 (PMC5395909; doi:10.1186/s12955-017-0642-8)
Supplement: Supplementary file 1 — Changed scores of measures in three groups. (DOCX 17 kb) [file 12955_2017_642_MOESM1_ESM.docx]

**Additional file 1: Changed scores of measures in three groups**

| measures | Improved patients Mean ± SD | Stable patients  Mean ± SD | Worsening patients  Mean ± SD |
| --- | --- | --- | --- |
| GDS | −1.19 ± 3.33 | 1.13 ± 6.67 | 1.94 ± 5.54 |
| MDS-UPDRS Part II | −2.38 ± 3.88 | 0.38 ± 3.70 | 3.21 ± 5.05 |
| MDS-UPDRS Part III | −12.63 ± 9.05 | 1.50 ± 2.40 | 15.62 ± 6.67 |
| SF-36 total scores | 4.58 ± 15.52 | −3.79 ± 14.52 | −6.17 ± 14.24 |
| PDQ-39 SI | −2.59 ± 7.02 | 0.85 ± 7.38 | 6.00 ± 10.98 |

GDS, Geriatric Depression Scale; MDS-UPDRS, Movement Disorder Society Revision of the Unified Parkinson's Disease Rating Scale; SF-36, 36-item Short Form Health Survey; PDQ-39, 39-item Parkinson’s Disease Questionnaire Single Index; SI, summary index; SD, standard deviation.
