# Supplementary material for: Responsiveness of the short-form health survey and the Parkinson’s disease questionnaire in patients with Parkinson’s disease
Source: Health Qual Life Outcomes. 2017 Apr 18;15:75. doi: 10.1186/s12955-017-0642-8 (PMC5395909; doi:10.1186/s12955-017-0642-8)
Supplement: Supplementary file 2 — Internal responsiveness of HRQoL in the stable patient group. (DOCX 20 kb) [file 12955_2017_642_MOESM2_ESM.docx]

**Additional file 2: Internal responsiveness of HRQoL in the stable patient group**

| HRQoL  domains | Stable (n = 24) | | | | |
| --- | --- | --- | --- | --- | --- |
|  | Baseline  mean  (SD) | Follow-up  mean  (SD) | ES | SRM | *p* |
| **SF-36** |  |  |  |  |  |
| Physical  functioning | 78.54  (18.97) | 77.50  (15.67) | −0.05 | −0.07 | 0.609 |
| Role-physical | 54.17  (30.99) | 55.21  (39.00) | 0.03 | 0.03 | 0.946 |
| Bodily pain | 79.13  (21.37) | 71.88  (18.20) | −0.34 | −0.33 | 0.164 |
| General health | 51.50  (21.32) | 40.79  (20.56) | −0.50 | −0.59 | 0.005 |
| Mental health | 70.33  (24.29) | 69.50  (24.68) | −0.03 | −0.03 | 0.553 |
| Role-emotional | 63.89  (37.96) | 52.78  (40.43) | −0.29 | −0.29 | 0.164 |
| Social functioning | 76.04  (24.15) | 77.08  (19.03) | 0.04 | 0.04 | 0.690 |
| Vitality | 55.83  (27.09) | 54.38  (19.69) | −0.05 | −0.06 | 0.681 |
| Total scores | 66.18  (18.54) | 62.39  (16.09) | −0.20 | −0.26 | 0.024 |
| **PDQ-39** |  |  |  |  |  |
| Mobility | 11.77  (16.69) | 12.40  (13.03) | −0.04 | −0.08 | 0.694 |
| ADL | 10.42  (14.01) | 12.33  (11.09) | −0.14 | −0.11 | 0.268 |
| Emotional well-being | 13.19  (9.65) | 15.28  (15.13) | −0.22 | −0.18 | 0.457 |
| Stigma | 20.05  (22.87) | 16.41  (18.96) | 0.16 | 0.23 | 0.224 |
| Social support | 9.90  (20.22) | 9.37  (15.06) | 0.03 | 0.04 | 0.591 |
| Cognitions | 21.09  (12.88) | 20.83  (13.50) | 0.02 | 0.02 | 0.870 |
| Communication | 7.99  (9.67) | 10.76  (12.65) | −0.29 | −0.19 | 0.394 |
| Bodily discomfort | 18.75  (19.39) | 22.57  (0.38) | −0.20 | −0.25 | 0.343 |
| SI | 14.15  (8.91) | 14.99  (8.36) | −0.10 | −0.12 | 0.310 |

SF-36, 36-item Short Form Health Survey; PDQ-39, 39-item Parkinson’s Disease Questionnaire Single Index; ADL, activities of daily living; SI, summary index; SD, standard deviation; ES, effect size; SRM, standardized response mean.
